# Use of chlorine dioxide to sterilize medium for tissue culture of potato

**DOI:** 10.1038/s41598-019-46795-4

**Published:** 2019-07-15

**Authors:** Yongbo Duan, Han Zhang, Mengchu Sun, Fenglan Zhao, Tao Xue, Jianping Xue

**Affiliations:** Key Laboratory of Resource Plant Biology of Anhui Province, College of Life Sciences, Huaibei Normal University, Huaibei, 235000 China

**Keywords:** Agricultural genetics, Molecular engineering in plants

## Abstract

*In vitro* cultured seedlings or microtubers are the major starting materials for the production of potato. Currently, seedlings are cultured in media sterilized by autoclaving, which, however, consumes more electricity and takes longer for sterilization, and also requires high temperature-tolerant vessel materials. In order to identify alternative methods of sterilizing culture conditions, the disinfection effects of chlorine dioxide (CD) at 88.0, 29.3, 17.6, 12.6 and 8.8 μM were evaluated in potato medium and vessels. The ≥12.6 μM gaseous CD effectively disinfected vessel through a 30-min fumigation process, and its aqueous solution disinfected potato medium efficiently as well. In presence of 12.6 μM CD in the medium, the potato seedlings had similar morphological features as those grown on autoclaved medium, with some exceptions. The use of 12.6–29.3 μM aqueous CD to sterilize the medium increased antioxidant enzyme activities in potato seedlings, while the use of higher concentration decreased antioxidant enzyme activity levels. SSR analysis did not reveal significant molecular differences in potato seedlings cultured between autoclaved and CD-sterilized medium. In addition to this, CD-sterilized medium induced potato microtuber formation at a similar rate as autoclaved medium. In summary, using CD to sterilize potato medium and vessels did not compromise the growth of seedlings and microtuber induction. This study provides an economical and simplified sterilization method for media used to culture potato plantlets, and this can improve energy use of the large-scale tissue culture industry.

## Introduction

Potato (*Solanum tuberosum L*.) is a multipurpose crop and is the 4^th^ most produced crop across the world, following the staple food grains wheat (*Triticum aestivum*), maize (*Zea mays*) and rice (*Oryza sativa*), possessing strategic importance to guarantee food security worldwide. In 2016, People’s Republic of China, which has 20% of the global population, issued *Guidance on Promoting the Development of the Potato Industry*^1^, which outlined the plan to extend potato cultivation acreage up to 17 million acres by 2020.

Two major limitations for potato seed production are diseases and the low multiplication rate^2,3^. *In vitro* cultured seedlings, or microtubers, which are pathogen-free and genetically identical to field grown seedlings, together with their shortened growth period, provide an effective solution to address the limitations in mass-producing potato seeds^4^. Robust potato seed propagation protocols have been developed and applied at the commercial scale, and this was achieved by optimizing illumination condition, medium composition, and hormone combinations, as summarized by Dobránszki *et al*.^5^.

The medium and vessels used in culturing potato seeds must be effectively sterilized to inactivate microbial populations without compromising the growth of the potato seedlings. Currently, media and vessels are sterilized by autoclaving at 121 °C for 20–30 min under high pressure (102.9 kPa). However, autoclaving has some disadvantages that impede the wide use of tissue culture methods to culture potato. The cost of electricity to autoclave materials is always an important contributor to total medium costs^6,7^, although there may be some proportional differences among countries. Autoclaving also requires the use of vessels that are tolerant to high temperatures and pressure. Transparent, high quality materials allow for better illumination and plant growth conditions, but these materials are often intolerant to high heat. Instead, materials commonly used for autoclaving are not transparent and hinder growth of plants due to reduced illumination. Moreover, autoclaving may degrade some chemicals in the culture media, or generate harmful substances, such as sucrose-derived aldehydes and phenols that can result from the catalysis of FeNa-EDTA during the high temperature sterilization process^8^. For these reasons, different sterilization methods have been tested in plant tissue culture, such as the microwave oven^9^, plant essential oils^10^ and chemicals. Chemical sterilization is advantageous in that it is more cost effective than autoclaving^11^. Accordingly, a number of chemicals, including diethyl pyrocarbonate^12^, chlorine dioxide^13–16^, peracetic acid^13^, sodium hypochlorite^17–19^, and hydrogen peroxide^19^, have been used to sterilize plant medium culture. However, chemical sterilization of media has not been tested in potato tissue culture, despite the need for improved conditions to reduce the cost of producing microtubers.

Here, we present the application of chlorine dioxide (CD) to disinfect potato culture medium and a comprehensive evaluation of seedlings cultured in media sterilized with CD at the phenotypic, physiological and molecular levels. This method of sterilization may facilitate the use of pathogen-free materials in potato production by making the process economical, environmentally safe and simple.

## Results

### Sterilization of vessels and medium with CD

Vessels were sterilized with CD by fumigating materials with gaseous CD in an airtight incubator. In all CD fumigation treatments tested, contamination was only observed in the vessels fumigated with the lowest concentration (8.8 μM). Contamination rates at 8.8 μM gaseous CD were 100%, 52% and 8%, at 10, 20 and 30 min fumigation treatments, respectively. This indicates that sterilization of vessels requires relatively longer fumigation at lower CD concentrations. The effective concentration of gaseous CD to sterilize vessels using fumigation was minimum of 12.6 μM for 10 min, where longer or high concentrations of treatments were more effective. These results demonstrate that fumigating vessels with CD effectively inactivates microbes, similar to the results of glass surface sterilization via CD fumigation reported previously^20^.

**Figure 1:**
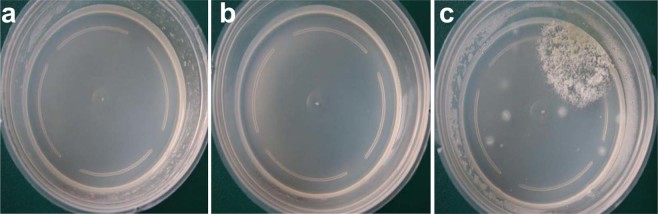
The effects of different sterilization methods on culture vessels and media 15 days after preparation. (**a)** An autoclaved vessel containing autoclaved medium, (**b)** a 12.6 mM gaseous CD fumigated vessel containing 12.6 mM aqueous CD sterilized medium, and (**c)** an autoclaved vessel containing non-sterilized medium. MS basal salts supplemented with 3% sucrose and 0.7% agar was used as the medium.

Similar to the sterilization of vessels, media sterilized with 8.8 μM CD was contaminated, while media treated with 12.6 μM or higher concentrations of CD, as well as media that was autoclaved, remained axenic (Table 1, Fig. 1). Results were similar when plants were cultured in the media, where only those sterilized with 8.8 μM CD became contaminated. Moreover, there was no excessive moisture at the surface of media sterilized with CD and walls of the vessels (Fig. 1), thus it could be used for experimentation immediately after solidification. Autoclaving involves subjecting materials to high temperature and high pressure conditions for approximately 20 mins, while CD is incorporated into the media and can continue to sterilize materials throughout the experiment through the slow release of CD into the microenvironment of vessels.

**Table 1:** Disinfection of media using different methods.

Treatments	CK	Gaseous chlorine dioxide μM				
		88	29.3	17.6	12.6	8.8
With explants	100%	100%	100%	100%	100%	0
Without explants	100%	100%	100%	100%	100%	0

CK stands for media autoclaved at 121 °C for 20 min at 102.9 kPa. All sterilized media was poured into 12.6 μM gaseous CD-fumigated vessels. Twenty vessels were included for each treatment. The data were recorded 15 days later.

The presence of CD, however, may impose an environmental stress and oxidizing effect on cultured plants. Therefore, suitable concentrations of CD that can sterilize materials and facilitate the growth of plants need to be determined.

### Morphological characters of potato seedlings cultured on CD-sterilized medium

**Figure 2:**
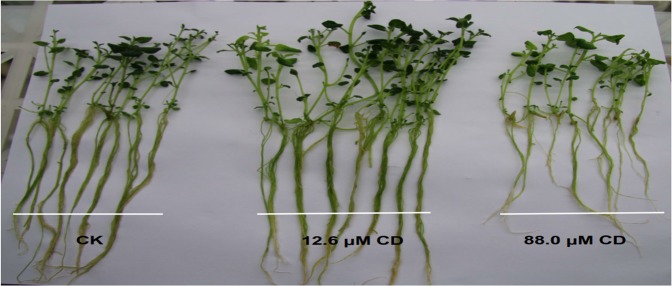
Seedlings of potato cultivar Zhong 5 cultured for 25 d in MS medium sterilized by autoclaving (left), and 12.6 (middle) and 88.0 μM aqueous CD (right).

To investigate the potential influence of CD on potato seedlings, three potato cultivars were cultured on media sterilized with 12.6, 17.6, 29.3 and 88.0 μM aqueous CD. Seedlings propagated on medium sterilized with various concentrations of CD showed differences in morphological indices, including the plant height, root length, branches and biomass (Table 2; Fig. 2). There were cultivar-specific morphological responses on different media. For plant height, Zhong 5 seedlings did not vary between autoclaved medium and 12.6, 17.6 and 29.3 μM CD-sterilized medium (*P* > 0.05), while plants cultured on 88.0 μM CD-sterilized medium were significantly shorter (*P* < 0.05). Hua 1 seedlings had similar height between autoclaved medium and 12.6 μM CD-sterilized medium (*P* > 0.05), and Hua 1 seedlings were significantly shorter when cultured in media that had been sterilized with higher CD concentrations (*P* < 0.05). Interestingly, A.R.I. seedlings had similar height on autoclaved and CD-sterilized media. Mean plant weight, root length and number of branches showed similar trends as plant height to different media. Taking the results from different cultivars into consideration, plants showed similar morphological features on media sterilized with 12.6 μM CD and autoclaved media (*P* > 0.05). These results indicate that the most appropriate concentration of aqueous CD for potato medium sterilization is 12.6 μM.

**Table 2:** Morphological characteristics of three potato cultivars in media sterilized with different treatments.

Variety	Sterilization method	Plant height (cm)	Per plant weight (mg)	Root length (cm)	Per plant branches
Zhong 5	CK	7.04 ± 0.23a	310.00 ± 48.01a	10.61 ± 1.88ab	5.99 ± 0.77ab
	88.0	6.21 ± 0.47b	164.00 ± 23.26b	6.10 ± 0.19c	2.79 ± 0.17c
	29.3	7.15 ± 0.34a	256.67 ± 35.12a	9.46 ± 0.12b	5.51 ± 0.31ab
	17.6	7.22 ± 0.40a	296.67 ± 37.86a	11.57 ± 1.72ab	5.22 ± 0.09b
	12.6	7.71 ± 0.30a	333.33 ± 56.86a	12.60 ± 1.70a	6.13 ± 0.55a
Hua 1	CK	8.08 ± 0.63a	153.33 ± 19.50ab	5.59 ± 0.26a	0.88 ± 0.13a
	88.0	6.33 ± 0.21c	86.00 ± 5.29c	4.01 ± 0.58b	0.85 ± 0.04a
	29.3	6.73 ± 0.17bc	95.00 ± 9.54c	4.40 ± 0.41b	0.84 ± 0.06a
	17.6	7.20 ± 0.31b	136.67 ± 15.28 b	4.67 ± 0.52b	0.85 ± 0.03a
	12.6	7.95 ± 0.35a	170.00 ± 10.00a	5.80 ± 0.43a	0.94 ± 0.08a
A.R.I.	CK	7.23 ± 1.05a	403.33 ± 25.17ab	13.52 ± 2.58ab	0.76 ± 0.08ab
	88.0	7.16 ± 0.51a	343.33 ± 30.55b	10.47 ± 1.62b	0.55 ± 0.07c
	29.3	7.17 ± 0.14a	350.00 ± 26.46b	12.35 ± 0.92ab	0.74 ± 0.05b
	17.6	7.42 ± 0.35a	426.67 ± 40.41a	14.37 ± 2.37ab	0.84 ± 0.05ab
	12.6	7.69 ± 0.43a	433.33 ± 15.28a	16.53 ± 1.63a	0.89 ± 0.09a

Data within one column followed by different lowercase letters indicate significant differences by Duncan’s multiple range test (*P* < 0.05).

### Physiological indices of potato seedlings

To test for the potential effects of different media on antioxidant enzyme activity in potato seedlings, the activities of SOD, POX and CAT were measured in potato seedlings cultured on autoclaved and CD-sterilized media (Table 3). SOD activity in Zhong 5 seedlings cultured on 12.6 μM CD-sterilized media was similar with those cultured on autoclaved media. The SOD activity in 12.6 μM CD-sterilized was lower than seedlings cultured on 17.6 and 29.3 μM CD-sterilized media and significantly higher than seedlings cultured on 88.0 μM CD-sterilized media (*P* < 0.05). Hua 1 seedlings on 12.6 μM CD-sterilized media had similar SOD activity as autoclaved medium and SOD activity was significantly higher in 12.6 μM media compared to other treatments (*P* < 0.05). In A.R.I. seedlings, autoclaved and 12.6 μM CD-sterilized media had similar SOD activity levels, which were significantly lower than seedlings cultured on 17.6 and 29.3 μM CD-sterilized media and higher than seedlings cultured on 88.0 μM CD-sterilized media (*P* < 0.05). Similar trends were observed in POX and CAT (Table 3). Taken together, potato seedlings cultured on 88.0 μM CD sterilized media had significantly reduced POX, CAT and SOD activities compared to autoclaved media (*P* < 0.05), while media sterilized with 12.6 μM, 17.6 μM, and 29.3 μM CD showed equal or higher antioxidant activities compared to autoclaved media.

**Table 3:** Physiological indices of potato plantlets cultured on different media.

Variety	Sterilization method	SOD (U/g·FW)	POX (U/g·FW)	CAT (μmol/g·FW)	MDA (μmol/g·FW)
Zhong 5	CK	326.67 ± 29.19b	53.28 ± 6.15 b	7.92 ± 0.81a	3.87 ± 0.27c
	12.6	323.07 ± 13.22b	52.20 ± 4.55b	7.78 ± 0.96a	3.83 ± 0.18c
	17.6	406.08 ± 23.79a	60.87 ± 6.77 ab	8.97 ± 0.89a	4.03 ± 0.21c
	29.3	426.49 ± 22.33a	69.34 ± 7.60a	9.34 ± 1.07a	5.82 ± 0.53b
	88.0	167.01 ± 14.41c	39.43 ± 5.25c	5.68 ± 0.46b	7.02 ± 1.13a
Hua 1	CK	183.28 ± 17.61b	43.35 ± 6.21abc	9.31 ± 0.96a	0.89 ± 0.10d
	12.6	177.20 ± 9.36b	41.17 ± 5.84bc	9.20 ± 0.30a	1.13 ± 0.27d
	17.6	213.22 ± 15.40a	55.33 ± 8.84a	9.56 ± 0.23a	1.66 ± 0.14c
	29.3	242.42 ± 18.33a	53.33 ± 5.94 ab	9.43 ± 0.54a	2.24 ± 0.45b
	88.0	66.43 ± 6.40d	35.67 ± 5.25c	8.01 ± 0.13b	3.12 ± 0.34a
A.R.I.	CK	310.32 ± 23.20c	49.80 ± 6.79c	6.60 ± 1.18c	1.05 ± 0.16c
	12.6	317.50 ± 34.78c	54.47 ± 6.40bc	6.30 ± 0.56c	1.15 ± 0.18c
	17.6	402.90 ± 35.35b	64.37 ± 8.98ab	8.43 ± 0.50b	2.09 ± 0.13b
	29.3	461.33 ± 20.40a	69.07 ± 9.55a	9.87 ± 0.83a	2.36 ± 0.07b
	88.0	200.87 ± 14.60d	44.90 ± 3.86c	4.47 ± 0.32d	3.64 ± 0.26a

Data within one column followed by different lowercase letters were significantly different by Duncan’s multiple range test (*P* < 0.05).

Some differences were observed in MDA accumulation in seedlings cultured on media treated with different sterilization conditions (Table 3). Seedlings of all three varieties grown on media sterilized with 88.0 μM CD accumulated more MDA compared to autoclaved media (*P* < 0.05), and seedlings cultured on 12.6 μM CD-sterilized media showed similar MDA levels as autoclaved media (*P* > 0.05).

The differences in antioxidant enzyme levels observed in seedlings cultured on higher concentrations of CD suggest that excessive CD can create a stressful environment for potato seedlings, therefore the sterilizing effect and potential negative impact on potato seedlings must be balanced when choosing CD concentrations for sterilizing media. Based on the physiological performance of seedlings, the use of 12.6 μM CD appears to cause the least stressful environment for seedlings of three potato cultivars.

### Detection of genomic variation in potato seedlings via SSR

To investigate whether the presence of CD in the media lead to genomic variation in potato seedlings, 12 primer pairs^21^ were used to amplify SSR regions of the genomic DNA of potato cultured on medium sterilized with different methods.

**Figure 3:**
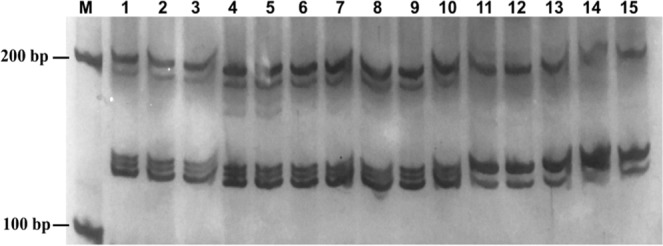
SSR pattern of primer pairs STI0003 on potato seedlings cultured with media that were sterilized by autoclaving and 12.6, 17.6, 29.3 and 88.0 μM CD. (**M**) DL 2000 DNA marker; (**1–5)** Zhong 5, (**6–10)** Hua 1, (**11–15)** A.R.I. The loading order for each cultivar is (**1, 6, 11**) autoclaving, and (**2, 7, 12**) 12.6, (**3, 8, 13**) 17.6, (**4, 9, 14**) 29.3 and (**5, 10, 15**) 88.0 μM CD.

Each primer pair resulted in cultivar-specific PCR band patterns and these patterns were preserved in samples cultured on the different media (Figs 3, S1). Of all the amplifications, only one polymorphic band (STI0012) was observed in Zhong 5 and one (SSR0707) observed in Hua 1 seedlings cultured on medium sterilized with 29.3 and 88 μM CD (Table 4). The results suggest that media containing 12.6 and 17.6 μM CD do not cause genetic variations in the potato genome.

**Table 4:** Genomic variation of cultured seedlings as determined with SSR primers.

Primer	Zhong 5		Hua 1		A.R.I.	
	No. of bands	No. of polymorphic bands	No. of bands	No. of polymorphic bands	No. of bands	No. of polymorphic bands
STM2022	4	0	5	0	5	0
STI0012	6	1	6	0	6	0
SSR0387	4	0	8	0	6	0
STG0016	8	0	8	0	9	0
SSR0707	4	0	4	1	3	0
STI0032	9	0	14	0	10	0
SSR0675	6	0	6	0	3	0
PM0938	2	0	2	0	3	0
STI0003	5	0	5	0	3	0
STG0025	3	0	3	0	3	0
PM0890	2	0	2	0	3	0
PM0936	4	0	3	0	3	0

### Microtuber induction

**Figure 4:**
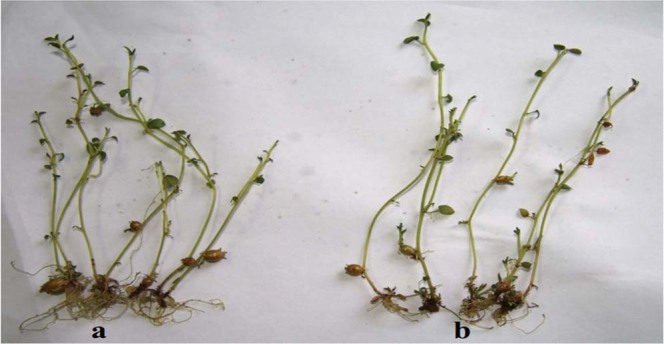
Microtubers of Hua 1 induced in medium sterilized with (**a)** autoclaved medium and (**b)** medium sterilized with 12.6 μM aqueous CD. Pictures were taken 60 days after culture.

**Figure 5:**
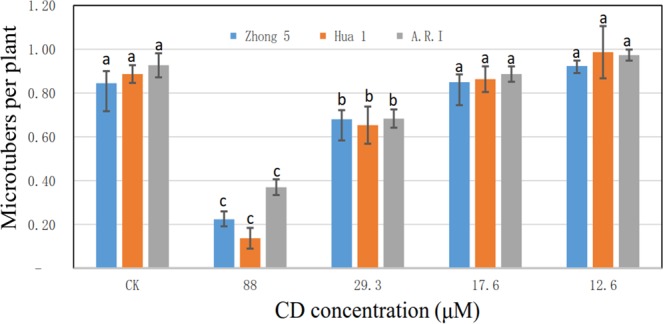
Induced microtubers cultured on media sterilized with autoclaving or various concentrations of aqueous CD for 60 days. Colored bars represent different cultivars where blue is Zhong 5, orange is Hua 1 and gray is A.R.I. Different lowercase letters indicate statistical significance at *P* < 0.05 for each cultivar.

Microtubers were induced by increasing the sucrose concentration of autoclaved and CD-sterilized media to 8% and applying them to nodal segments. Sixty days later, microtubers were collected from seedlings (Fig. 4). For Hua 1, media sterilized with 12.6 and 17.6 μM CD had comparable microtuber induction levels compared to autoclaved media (*P* > 0.05), while media sterilized with 29.3 and 88.0 μM CD did not yield many microtubers (Fig. 5) (P < 0.05). The number of per plant microtubers induced from autoclaved, 12.6 and 17.6 μM CD-sterilized medium was between 0.8–1.0, and it decreased to 0.7 and 0.2 in 29.3 and 88.0 μM CD-sterilized media, respectively. Zhong 5 and A.R.I. plantlets showed similar patterns of microtuber induction in response to the different media (Fig. 5).

In order to induce microtubers, samples need to be cultured for up to 60 days. Interestingly, some contamination was observed in media sterilized with 12.6 μM CD after 60 days of culture. These results suggest that microtuber induction should be conducted using 17.6 μM CD sterilized media, which yielded similar results to autoclaved media.

## Discussion

The high cost of electricity during autoclaving has limited the use of *in vitro* plant cultures at a commercial scale. Thus, the development of an economical and simple method to sterilize plant culture media is of great practical significance to culture potato seedlings at an industrial-scale.

CD is an environmentally safe disinfectant that does not release toxic substances while sterilizing. CD has been applied broadly, including to disinfect tap water^22^, refresh agri-products^23^ and sterilize air^24,25^. In 2009, Cardoso successfully used CD to sterilize plant media to culture anthurium^13^. Afterwards, CD was applied to sterilize culture media for gerbera, and had similar sterilization efficiency as autoclaving, and resulted in similar or better growth of plants^14–16^. Our research group has evaluated the effectiveness of CD to disinfect explants to culture various plant species. Though CD can inactivate microbiota on plant explants, the viability of plants after CD-treatment was species-dependent^26^. Results from these recent studies indicate that the concentration of CD used to sterilize plant tissue culture and its effects on plant explants need to be taken into consideration.

In the present study, we describe the application of CD to sterilize plant culture media and vessels, and evaluate the influence of various CD concentrations on potato seedlings. CD was generated through a chemical reaction between sodium chlorite and food grade hydrochloric acid^27^, where sodium chloride was the only byproduct. High concentrations of sodium chloride, however, are toxic to plant cultures. Different plant species vary in saline tolerance, therefore the effective concentration of CD that can maintain normal growth of explants needs to be tested for each species. Previously, the commercial solution Tecsa-Clor^®^, which contains CD, has been tested to sterilize gerbera culture media^14^. The generation of CD through a chemical reaction, as performed in this study, is convenient for gaseous or aqueous CD. Gaseous CD was employed to sterilize vessels for the first time and 12.6 μM CD was effective for sterilizing vessels after a 30-min fumigation process. Though high concentrations of gaseous CD (>10%) can be explosive^28–30^, the effective CD concentration for vessel sterilization is far lower than 10%, thus it does not pose a safety risk. Fumigating culture vessels with gaseous CD is a novel approach for vessel sterilization and allows for the repeated use of vessels that are not heat or pressure tolerant and therefore unsuitable for autoclaving.

As low as 12.6 μM aqueous CD was capable of sterilizing potato culture media. This concentration was lower than the concentration of sodium hypochlorite (NaOCl) used to sterilize media in previous studies^17,18,31^, but similar to the CD concentrations used in other plant species^13–16^. Another advantage of using CD for sterilization is the low cost; the prices of sodium chlorite and food grade hydrochloric acid are far lower than commercial sodium hypochlorite products^11^.

CD used to sterilize media can extend the sterilization effect, however, it is also a strong oxidizing agent that can cause stress to potato seedlings. Thus, the effect of CD on the growth of potato seedlings requires further systematic evaluation. In this study, the morphological indices, including plant height, root length, branches and biomass, decreased with increasing CD concentrations. Although some differences were observed among the three cultivars when cultured on CD-sterilized media, seedlings cultured on 12.6 μM CD-sterilized media were morphologically similar to those cultured on autoclaved media. In addition, microtuber induced on 17.6 μM CD-sterilized media maintained an aseptic niche for two months, which was similar to autoclaved media. The similar morphological features and rates of microtuber induction between autoclaved and CD sterilized media are in line with previous studies including Paris *et al*.^18^, while Teixeira *et al*.^17^ and Cardoso & Silva^14^ who reported that CD containing medium promoted the growth of plants. The differences in response to media treated with chlorine are likely species-specific.

Exposure to CD can result in antioxidative stress in longan fruit when it is used for shelf-life extension^32^. CD-sterilized media may affect the antioxidant enzyme activities of plants; however, previous studies have not investigated this effect. Compared with autoclaved medium, the activities of the antioxidant enzymes POX, CAT and SOD increased in seedlings cultured on media with ≤29.3 μM CD, and decreased significantly in media sterilized with 88.0 μM CD (*P* < 0.05). This is in accordance to the physiological responses of plants to other environmental stressors, such as hydrogen peroxide^33^, lower temperature^34^ and drought^35^. MDA, one of the end products of stress-induced lipoperoxidation of polyunsaturated fatty acids, is an important index that can indicate the function and integrity of the plant cell membrane^36–38^. Of the CD concentrations that were tested, plants grown in media sterilized by 12.6 or 29.3 μM CD had similar MDA levels as seedlings on autoclaved media, though there were some differences among three potato cultivars. These results suggest that potato cells adapt to low-level CD stress by raising antioxidant enzyme levels to scavenge free radicals, while high CD stress likely destroys antioxidation system and decrease the activity of antioxidative enzymes.

Clonal fidelity is a very important indicator to evaluate the quality of tissue culture seedlings. Cardoso & Silva^14^ observed the normal growth of tissue culture gerbera seedlings cultured on CD-sterilized media until fully grown. In this study, we employed simple sequence repeats (SSRs) to study potential genetic changes in potatoes cultured on CD-incorporated medium. Most of the primers exhibited identical patterns when cultured on autoclaved and CD-sterilized media. This suggests that CD-sterilized media does not induce genomic variation in potato cultures.

In conclusion, culture vessels were successfully sterilized by fumigating materials with 12.6 μM gaseous CD for 30 min at room temperature, and potato culture media was sterilized by the addition of 12.6 μM aqueous CD for seedling multiplication and 17.6 μM aqueous CD for microtuber induction. The cultured potatoes were not different from those generated on autoclaved media at the morphological, physiological and molecular levels. Our results confirmed that CD can be used to effectively sterilize potato culture media and vessels, which may facilitate the production of pathogen-free seedlings and microtubers in a simple and economical manner.

## Methods

### Plant materials

The tissue culture seedlings of the potato cultivars Zhong 5, Hua 1 and A.R.I were provided by Prof. Jun Liu at the Huazhong Agricultural University, P.R. China. Seedlings were propagated through nodal cuttings, with each cutting having one axillary bud. Nodal cuttings were placed on MS basal medium^39^ containing 3% sucrose and 0.6% agar, and without exogenous growth regulators (pH 5.8). The nodal cuttings were incubated at 25 ± 1 °C and ≈60% relative humidity under an illumination scheme of 16 h/8 h (light/dark) with a light intensity of 50 μmol m^−2^ s^−1^. Three weeks after culturing cuttings on MS media, the micropropagated seedlings were sheared into nodal cuttings and used for further tests.

### Reagents

MS basal salts were purchased from Jiafeng Articles for Horticulture Shanghai Co., Ltd. (Shanghai, China), and other chemicals used in this study were purchased from Sigma (Saint Louis, MO, USA).

### Generation of CD

CD was generated by the chemical reaction between sodium chlorite (NaClO_2_) and hydrochloric acid (HCl) at room temperature through the following formula:

$${{\rm{5NaClO}}}_{{\rm{2}}}+{\rm{4HCl}}={{\rm{4ClO}}}_{{\rm{2}}}+{\rm{5NaCl}}+{{\rm{2H}}}_{{\rm{2}}}{\rm{O}}$$

To achieve this, sodium chlorite was dissolved in ddH_2_O and then mixed with food grade hydrochloric acid in a capped volumetric flask. Given sufficient hydrochloric acid, one gram of sodium chlorite can generate 594.7 mg of aqueous CD, which can be used to create a stock concentration of 8.8 mM at 1 L with ddH_2_O. The stock solution was stable for use for two weeks in a brown airtight glass bottle at 4 °C, and was diluted to working concentration before use. To generate gaseous CD, sodium chlorite was directly mixed with sufficient hydrochloric acid to produce gaseous CD. The concentration of gaseous CD was adjusted according to the volume of the airtight room.

### Medium preparation and sterilization

MS basal salts and sucrose were dissolved and stirred in ddH_2_O containing CD at 60% medium volume in an airtight flask. Agar was dissolved in ddH_2_O at the remaining 40% medium volume and heated in a microwave oven until the solution became transparent. The agar solution was poured into the rest of the medium and stirred for 20 min. The pH of the medium was adjusted to 5.8 using potassium hydroxide. Vessels were sterilized by fumigation with gaseous CD in an airtight room and the sterilized medium was poured into sterilized vessels in a laminar flow hood.

Under airtight condition, various concentrations (88.0, 29.3, 17.6, 12.6 and 8.8 μM) of gaseous CD were freshly generated to fumigate by uncapping vessels and caps for 10, 20 and 30 mins. The sterilization effect was detected by pouring autoclaved MS basal medium into the fumigated vessels in a laminar flow hood and incubating at 25 °C for 15 d. For medium sterilization, various concentrations (88.0, 29.3, 17.6, 12.6 and 8.8 μM) of aqueous CD were used to prepare media. Sterilized media was poured into CD-fumigated vessels in a laminar flow hood and incubated at 25 °C. For the sterilization of both vessels and medium, autoclaved vessels poured with autoclaved medium were used as control, and the sterilization effect was observed and recorded after 15 days.

### Potato seedling multiplication and microtuber induction

Seedling multiplication was induced using hormone-free MS basal medium supplemented with 3% sucrose and 0.6% agar to obtain microtubers. The nodal cuttings were incubated at 25 ± 1 °C and ≈60% relative humidity conditions, under an illumination scheme of 8 h/16 h (light/dark) with a light intensity of 50 μmol m^−2^ s^−1^. The control medium was autoclaved at 121 °C, 1 kg/cm^2^ for 20 min.

### Morphological indices

Twenty-five days after culture, morphological indices of the seedlings were measured, including plant height, root number, root length, branches and fresh biomass. Only primary roots were counted for root number. Ten randomly selected seedlings were recorded and averaged for each index per treatment conditions.

### Measurement of physiological indices

The superoxide dismutase (SOD), peroxidase (POX), catalase (CAT) and malondialdehyde (MDA) were measured following Zhang *et al*.^40^. Each treatment was conducted in triplicate and each replicate was pooled from three to five seedlings.

To measure SOD activity 0.5 g of seedlings from each treatment was ground in 10 ml of precooled PBS then centrifuged at 1000 r/min at 4 °C for 20 min. The supernatant was removed as the SOD extract. The reaction mixture contained 1.5 ml of 50 mM phosphate buffer solution (PBS, pH 7.0), 0.3 ml of 100 μM EDTA-Na_2_, 0.3 ml of 130 mM MET, 0.3 ml of 750 μM NBT, 0.5 ml of ddH_2_O, 0.1 ml of SOD extract and 0.3 ml of 20 μM riboflavin. Two blank controls were prepared by using 0.1 ml ddH_2_O to substitute for the SOD extract in the reaction mixture. One blank control was immediately placed in the dark once prepared, while the other blank control, together with the reaction samples, was illuminated with a fluorescent lamp for 15 min. Using the control incubated in the dark as a blank, the *A*_560_ of all reactions was measured using a spectrophotometer.

To measure POX activity 1 g of seedlings from each treatment was ground in 5 ml of 20 mM PBS in a mortar and then centrifuged at 4000 r/min at 4 °C for 15 min. The supernatant was removed as the POX extract. The supernatant volume was increased to 8 ml with PBS. A reaction mixture solution was prepared by adding 28 μl of guaiacol and 19 μl of 30% hydrogen peroxide into 50 ml of 100 mM PBS. Before detection, 200 μl of POX extract was mixed with 3 ml of phosphate buffer mixture, and used to measure the *A*_470_ at 30 s intervals.

To measure CAT activity 2 g of seedlings from each treatment were ground in 10 ml of acetone in a mortar and then centrifuged at 10000 r/min at 4 °C for 10 min. The supernatant volume was increased to 3 ml with acetone. This solution was mixed with 3 ml of solvent [CCl_4_:CHCl_3_ = 3:1], followed by mixing with 5 ml ddH_2_O. The mixture was centrifuged at 5000 r/min at 4 °C for 1 min, yielding the supernatant as the CAT extract. For CAT measurement, 1 ml CAT extract was mixed with 2 ml of reaction solution and used to measure *A*_560_ at 30 s intervals against a ddH_2_O blank.

To measure MDA content 1 g of seedlings from each treatment was ground in 5 ml of 10% trichloroacetic acid (TCA) in a mortar and then centrifuged at 4000 r/min at 4 °C for 10 min. The supernatant was removed as the MDA extract. For MDA measurements, 2 ml of MDA extract was mixed with 2 ml of 0.6% 2-thiobarbituric acid (TBA), then heated in a boiling water bath for 15 min. The solution was centrifuged at 4000 r/min for 15 min and then cooled to room temperature. The *A*_532_ and *A*_450_ were measured using a blank of equal volume of a mixture of ddH_2_O and 0.6% TBA.

### Detection of genomic variation via simple sequence repeats

To detect whether CD-sterilized medium led to genomic variation in potato plants, 12 pairs of simple sequence repeats (SSR) primers (Supplementary Table S1) spanning 12 chromosomes^14^ were used to amplify genomic DNA from plants cultured with media sterilized under different conditions. Genomic DNA isolation and PCR amplification were performed following previous publications^41^. The PCR components were denatured at 94 °C for 5 min, followed by 35 cycles of 94 °C 30 s, annealing at various temperatures (Supplementary Table S1) for 30 s and 72 °C 45 s, and a final extension at 72 °C for 5 min. The PCR product was detected by 8% SDS-PAGE gel electrophoresis and observed under a gel imaging system.

### Data analysis

All experiments were carried out in triplicate and data are presented as mean ± SD. The experimental treatments were compared using an analysis of variance (ANOVA) with a *P* value at or below 0.05 for the significance cut-off. Statistical significance between mean values of the replicates was assessed using a Duncan’s multiple range test (DMRT). Statistical analysis was performed using SPSS 21.0.

## Supplementary information

Supplementary information

## Data Availability

The datasets supporting the conclusions and description are included within the article.

## References

[1] Li JD, Li XD, Wang SH (2017). Analysis on financialization of potato price fluctuation under background of developing potato as staple food. J Huazhong Agri Univ.

[2] Wang B (2011). Potato viruses in China. Crop Prot.

[3] Li YL (2017). Integrated miRNA and microRNA transcriptome analysis reveals miRNA regulation in response to PVA in potato. Sci Rep.

[4] Venkatasalam EP (2013). Development of low cost technology for *in vitro* mass multiplication of potato (*Solanum tuberosum* L.). Afr J Agr Res.

[5] Dobránszki J, Magyar-Tábori K, Hudák I (2008). *In vitro* tuberization in hormone-free systems on solidified medium and dormancy of potato microtubers. Fruit Veg Cereal Sci Biotechnol.

[6] IAEA-TECDOC. Low cost options for tissue culture technology in developing countries. *IAEA-TECDOC-1384*, AEA (2004).

[7] Manuel J (2013). Low cost tissue culture technology for the regeneration of some economically important plants for developing countries. International Journal of Agriculture Environment & Biotechnology.

[8] Schenk N, Hsiao K, Bornman CH (1991). Avoidance of precipitation and carbohydrate breakdown in autoclaved plant tissue culture media. Plant Cell Rep.

[9] Venturieri GA, Venturieri AR, Leopoldo G (2013). Sterilization of culture media for orchids using a microwave oven. In Vitro Cell Dev-Pl.

[10] Deein W, Thepsithar C, Thongpukdee A (2013). *In vitro* culture medium sterilization by chemicals and essential oils without autoclaving and growth of chrysanthemum nodes. World Acad Sci Engin Technol.

[11] Silva ALL, Brondani GE, Oliveira LS, Gonçalves NA (2013). Chemical sterilization of culture medium: a low cost alternative to *in vitro* establishment of plants. Sci For.

[12] Macek T, Král J, Vaněk T, Blažek J, Macková M (1994). Chemical sterilization of nutrient media for plant cell cultures using diethylpyrocarbonate. Biotechnol Tech.

[13] Cardoso JC (2009). Chemical sterilization of culture medium for anthurium *in vitro* culture. Pesqui Agropecu Bras.

[14] Cardoso JC, Silva JATD (2012). Micropropagation of gerbera using chlorine dioxide (ClO_2_) to sterilize the culture medium. In Vitro Cell Dev-Pl.

[15] Cardoso, J. C., Sheng Gerald, L. T. & Teixeira da Silva, J. A. Micropropagation in the Twenty-First Century. In: Loyola-Vargas, V. & Ochoa-Alejo, N. (eds) Plant Cell Culture Protocols. Methods in Molecular Biology, vol 1815. Humana Press, New York, NY, 17–31 (2018). 10.1007/978-1-4939-8594-4_2 29981112

[16] Cardoso JC, Imthurn ACP (2018). Easy and efficient chemical sterilization of the culture medium for *in vitro* growth of gerbera using chlorine dioxide (ClO_2_). Ornam Hortic.

[17] Teixeira SL, Ribeiro JM, Teixeira MT (2006). Influence of NaOCl on nutrient medium sterilization and on pineapple (*Ananas comosus*, cv *Smooth cayenne*) behavior. PCTOC.

[18] Pais AK (2016). Sodium hypochlorite sterilization of culture medium in micropropagation of *Gerbera hybrida* cv. *Essandre*. Afr J Biotechnol.

[19] Yanagawa T, Nagai M, Ogino T, Maeguchi R (1995). Application of disinfectants to orchid seeds, plantlets and media as a means to prevent *in vitro* contamination. Lindleyana.

[20] Morino H, Fukuda T, Miura T, Shibata T (2011). Effect of low-concentration chlorine dioxide gas against bacteria and viruses on a glass surface in wet environments. Lett Appl Microbiol.

[21] Song X (2016). SSR Analysis of genetic diversity among 192 diploid potato cultivars. Hort Plant J.

[22] Simon FX, Berdalet E, Gracia FA, España F, Llorens J (2014). Seawater disinfection by chlorine dioxide and sodium hypochlorite-A comparison of biofilm formation. Water Air Soil Poll.

[23] Park SH, Kang DH (2015). Antimicrobial effect of chlorine dioxide gas against foodborne pathogens under differing conditions of relative humidity. LWT-Food Sci Technol.

[24] Hsu CS, Huang DJ (2013). Disinfection efficiency of chlorine dioxide gas in student cafeterias in Taiwan. *J Air*. Waste Manage.

[25] Hsu CS, Lu MC, Huang DJ (2015). Disinfection of indoor air microorganisms in stack room of university library using gaseous chlorine dioxide. Environ Monit Assess.

[26] Duan YB (2016). Evaluation of aqueous chlorine dioxide for disinfecting plant explants. In Vitro Cell Dev-Pl.

[27] Mo ZB, Hu ST, Hu DD (2017). Experimental research on the manufacture of chlorine dioxide by sodium chlorite and hydrochloric acid at low concentration. J Shandong Univ.

[28] Gordon D, Carruthers BA, Theriault S (2012). Gaseous decontamination methods in high-containment laboratories. Applied Biosafety.

[29] Lowe JJ, Hewlett AL, Iwen PC, Smith PW, Gibbs SG (2013). Evaluation of ambulance decontamination using gaseous chlorine dioxide. Prehosp Emerg Care.

[30] Jia HQ (2013). Evaluation of gaseous chlorine dioxide fumigation for enclosed space decontamination. Milit Med Sci.

[31] Sawant RA, Tawar PN (2011). Use of sodium hypochlorite as media sterilant in sugarcane micropropagation at commercial scale. Sugar Tech.

[32] Chumyam A, Shank L, Faiyue B, Uthaibutra J, Saengnil K (2017). Effects of chlorine dioxide fumigation on redox balancing potential of antioxidative ascorbate-glutathione cycle in ‘Daw’ longan fruit during storage. Sci Hortic.

[33] Choudhary R, Saroha AE, Swarnkar PL (2012). Effect of abscisic acid and hydrogen peroxide on antioxidant enzymes in *Syzygium cumini* plant. J Food Sci Tech.

[34] Tsouvaltzis P, Brecht JK (2014). Changes in quality and antioxidant enzyme activities of bunched and topped radish (*Raphanus sativus* L.) plants during storage at 5 or 10 C. J Food Quality.

[35] Saeidnia F, Majidi MM, Mirlohi A, Soltan S (2017). Physiological and tolerance indices useful for drought tolerance selection in smooth bromegrass. Crop Sci.

[36] Davey MW, Stals E, Panis B, Keulemans J, Swennen RL (2005). High-throughput determination of malondialdehyde in plant tissues. Anal Biochem.

[37] Yamauchi Y, Furutera A, Toyoda Y, Tanaka K, Sugimoto Y (2008). Malondialdehyde generated from peroxidized linolenic acid causes protein modification in heat-stressed plants. Plant Physiol Bioch.

[38] Moustafa-Farag M (2015). Boron in I: activated antioxidant enzymes-reduced malondialdehyde concentration, and improved mineral uptake-promoted watermelon seedlings growth under boron deficiency. J Plant Nutr.

[39] Murashige T, Skoog F (1962). A revised medium for rapid growth and bioassays with tobacco tissue cultures. Phys Plant.

[40] Zhang, Z. L., Qu, W. J. & Li, X. F. Plant physiology experimental guidance [M]. Fourth edition. Beijing: Higher Education Press, 2009.

[41] Smith DS, Maxwell PW, De Boer SH (2005). Comparison of several methods for the extraction of DNA from potatoes and potato-derived products. J Agric Food Chem.

