# Supplementary material for: Use of chlorine dioxide to sterilize medium for tissue culture of potato
Source: Sci Rep. 2019 Jul 15;9:10232. doi: 10.1038/s41598-019-46795-4 (PMC6629699; doi:10.1038/s41598-019-46795-4)
Supplement: Supplementary file 1 — Supplementary information [file 41598_2019_46795_MOESM1_ESM.docx]

**Use of chlorine dioxide to sterilize medium for tissue culture of potato**

Yongbo Duan, Han Zhang, Mengchu Sun, Fenglan Zhao, Tao Xue^*^, Jianping Xue^*^

Key Laboratory of Resource Plant Biology of Anhui Province, College of Life Sciences, Huaibei Normal University, Huaibei, China 235000

* Corresponding authors. E-mails: xuetao_26@163.com to Tao Xue, [xuejp@163.com](mailto:xuejp@163.com) to Jianping Xue.


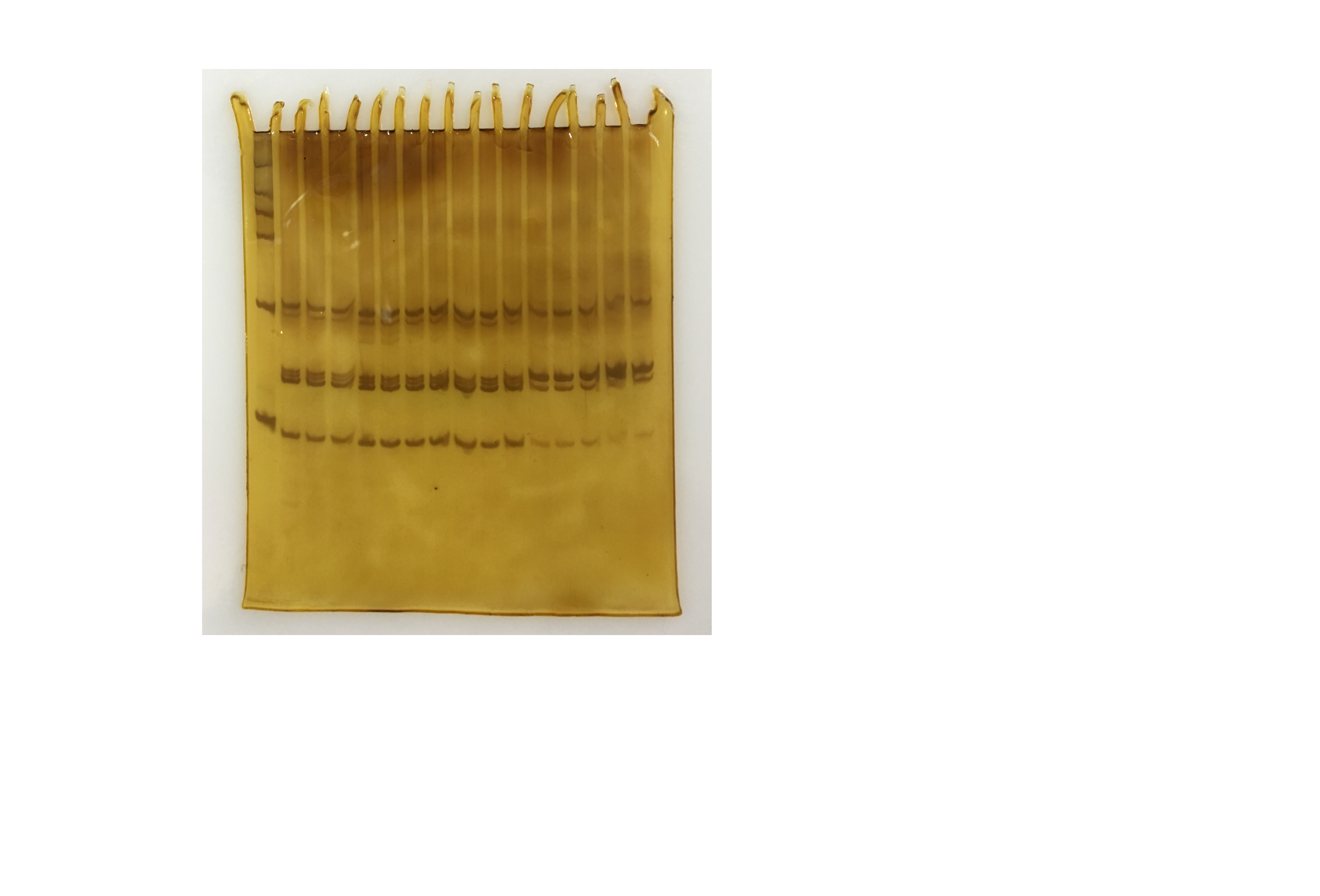


**Supplementary Figure S1** Original figure of SSR analysis with STI0003.
